# GYF-21, an Epoxide 2-(2-Phenethyl)-Chromone Derivative, Suppresses Innate and Adaptive Immunity via Inhibiting STAT1/3 and NF-κB Signaling Pathways

**DOI:** 10.3389/fphar.2017.00281

**Published:** 2017-05-22

**Authors:** Ran Guo, Yun-Fang Zhao, Jun Li, Yu-Fan Gu, Hui-Xia Huo, Shan-Shan Li, Yue-Lin Song, Zhi-Xiang Zhu, Peng-Fei Tu

**Affiliations:** Modern Research Center for Traditional Chinese Medicine, School of Chinese Materia Medica, Beijing University of Chinese MedicineBeijing, China

**Keywords:** multiple sclerosis, epoxide 2-(2-phenethyl)-chromone, innate immunity, adaptive immunity, STAT1/3 signaling pathway

## Abstract

Multiple sclerosis is a chronic inflammatory autoimmune disease of the central nervous system characterized by demyelinating plaques and axonal loss. Inhibition on over activation of innate and adaptive immunity provides a rationale strategy for treatment of multiple sclerosis. In the present study, we investigated the inhibitory effects of GYF-21, an epoxide 2-(2-phenethyl)-chromone derivative isolated from Chinese agarwood, on innate and adaptive immunity for revealing its potential to treat multiple sclerosis. The results showed that GYF-21 markedly inhibited the activation of microglia, and dendritic cells as well as neutrophils, all of which play important roles in innate immunity. Furthermore, GYF-21 significantly suppressed adaptive immunity via inhibiting the differentiation of naive CD4^+^ T cells into T helper 1 (Th1) and T helper 17 (Th17) cells, and suppressing the activation, proliferation, and IFN-γ secretion of CD8^+^ T cells. The mechanism study showed that GYF-21 evidently inhibited the activation of STAT1/3 and NF-κB signaling pathways in microglia. In conclusion, we demonstrated that GYF-21 can significantly inhibit innate and adaptive immunity via suppressing STAT1/3 and NF-κB signaling pathways, and has potential to be developed into therapeutic drug for multiple sclerosis.

## Introduction

As an organ-specific autoimmune disease, multiple sclerosis (MS) is manifested by chronic inflammatory demyelination of the central nervous system (CNS) and is one of the foremost causes of non-traumatic neurological disability in young adults (Goverman, 2009; Dendrou et al., 2015). CD4^+^ T cell-mediated autoimmunity has long been accepted as one of the most important aspects of MS pathogenesis, especially for the early initiation of diseases. T helper 1 (Th1) cells, characterized by the production of interferon-γ (IFN-γ), have been considered as the type of effector helper T cells that mediate the pathogenesis of MS; subsequently studies have indicated that interleukin 17 (IL-17)-producing T helper (Th17) cells are involved and play more important role in this pathogenesis than Th1 cells (Ogura et al., 2008; McGeachy et al., 2009; El-Behi et al., 2011; Lazarevic et al., 2011; Goldmann and Prinz, 2013; Martin et al., 2016). Myeloid innate immune cells, such as microglia, dendritic cells, and neutrophils, are prominent constituents of inflammatory infiltrates in the CNS during MS. These cells not only serve as antigen presenting cells for the reactivation of infiltrating myelin-reactive CD4^+^ T cells but also are thought to directly result in tissue damage through secretion of toxic factors (Wu and Laufer, 2007; Comabella et al., 2010; Starossom et al., 2012; Goldmann and Prinz, 2013; Jaillon et al., 2013; Nuyts et al., 2013; Steinbach et al., 2013; Strachan-Whaley et al., 2014).

However, only a few drugs, including interferon β, fingolimod, teriflunomide, dimethyl fumarate, glatiramer acetate, natalizumab, daclizumab, and mitoxantrone, are currently available for MS patients, and their clinical application is limited by poor curative effects and deleterious adverse effects (Hydleburg and D’Aversa, 2014; Rush et al., 2015; Torkildsen et al., 2016). Therefore, the identification of immunosuppressive drugs with new mechanism and lesser adverse effects to treat MS is very urgently needed.

**FIGURE 1:**
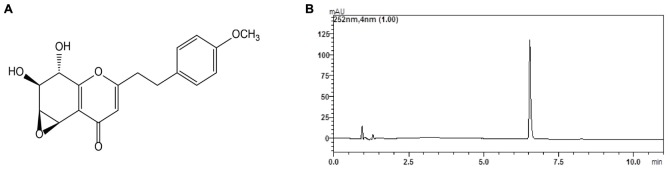
**Chemical structure** **(A)** and chromatogram **(B)** of GYF-21.

Chinese agarwood, a famous traditional Chinese medicine, has been used as a sedative, analgesic and digestive agent (Wu et al., 2012; Huo et al., 2015; Li et al., 2015; Zhu et al., 2016). Previously, we isolated dozens of 2-(phenethyl)-chromone derivatives from ethyl acetate extract of Chinese agarwood. In the preliminary bioactivity screening assays, we found that several 2-(phenethyl)-chromone derivatives significantly inhibited interleukin-6 (IL-6) production by LPS induced microglia, suggesting that these 2-(phenethyl)-chromone derivatives may have potential immunosuppressive activity (Huo et al., 2015; Zhu et al., 2016). Among these active 2-(phenethyl)-chromone derivatives, the compound (1a*S*, 2*S*, 3*S*, 7b*R*)-2, 3-dihydroxy-5-(4-methoxyphenethyl)-2, 3-dihydro-1a*H*-oxireno [2, 3-*f*] chromen-7 (7b*H*)-one (GYF-21, **Figure 1A**), which was firstly identified by Wu et al., showed strongest activity (Wu et al., 2012). In our previous study, we had demonstrated inhibitory effects of GYF-17, a 2-(phenethyl)-chromone derivative, on the activation of macrophages and microglia (data not shown) via mainly blocking STAT1/3 signaling pathways (Zhu et al., 2016). In view of the stronger inhibitory effects of GYF-21 on microglia and the important roles that STAT1/3 signaling pathways play in adaptive immune cells, we will investigate the inhibitory activity of GYF-21 on innate and adaptive immunity and underlying mechanisms, as well as reveal its potential to treat multiple sclerosis.

## Materials and Methods

### Reagents

GYF-21 was isolated from Chinese agarwood and the isolation procedure was described primarily (Huo et al., 2015). GYF-21 was dissolved at a concentration of 25 mM in dimethyl sulfoxide (DMSO). Cell Counting Kit-8 (CCK-8) was purchased from Beyotime, Co. (Shanghai, China). Lipopolysaccharide (LPS) from *Escherichia coli* O55: B5 was purchased from Sigma Chemicals, Co. (St. Louis, MO, United States). ELISA kits for determining TNF-α,IL-6, IL-1β, MCP-1, MIP-1α, IFN-γ, and IgG were purchased from R&D Systems (Minneapolis, MN, United States). The antibodies to p65, I-κB, p38, ERK1/2, JNK, STAT1, STAT3, and their phosphorylated forms were purchased from Cell signaling Technology (Beverly, MA, United States). Monoclonal antibodies (mAbs) conjugated to APC, APC, PE, FITC, PE, FITC, FITC, PE, PE-Cy7, Alexa Fluor 647, FITC, and PE (specific for Ly-6G, CD11c, CD11b, CD62L, CD69, CD25, CD80, CD86, CD4, CD8, IFN-γ, and IL-17A), and purified monoclonal antibodies (specific for CD3e, CD28, IFN-γ, and IL-4) were obtained from Becton Dickinson (San Diego, CA, United States). Magnetic bead isolation kit for mouse CD4^+^ T cells, naive CD4^+^ T cells and CD8^+^ T cells were purchased from Miltenyl Biotec (Bergisch Gladbach, Germany). Recombinant mouse GM-CSF, IL-4, IL-12, IFN-γ, IL-6, and TGF-β were purchased from PeproTech (Rocky Hill, NJ, United States).

### Isolation and Structural Identification of GYF-21

The air-dried agarwood powder (6.9 kg) was refluxed with 95% EtOH (3 h × 2.5 h). After removal of ethanol by evaporation, the residues (3.4 kg) were suspended in H_2_O and extracted with petroleum ether and ethyl acetate (EtOAc). The EtOAc fraction (400 g) was subjected to silica gel column chromatography (CC) and eluted with Petroleum ether–EtOAc (from 8:1 to 1:1) and CHCl_3_–CH_3_OH (from 20:1 to 1:3) to afford seven fractions (by TLC). Fraction 5 (86 g) was subjected to silica gel CC and eluted with CH_2_Cl_2_–CH_3_OH (from 80:1 to CH_3_OH) to give four fractions: 5a-5d. Fraction 5b (18 g) was subjected to CC and eluted with CH_2_Cl_2_–CH_3_OH (8:1), then introduced onto RP-ODS column and eluted with CH_3_OH–H_2_O (40:60). The stream was subjected to CC again and eluted with CH_2_Cl_2_–CH_3_O CH_3_ (10:1) to give GYF-21 (12 mg).

Spectroscopic and spectrometric analyses were employed for structural identification and purity analysis of GYF-21. ^1^H NMR and ^13^C NMR spectra were recorded on a Varian 500 MHz spectrometer (CA, United States) and deuterated dimethyl sulfoxide (DMSO-*d*_6_) was utilized as residual solvent. Chemical shifts were expressed in parts per million (ppm). Mass spectra were recorded on Shimadzu LCMS-IT-TOF platform (Tokyo, Japan).

### Animals

Male BALB/c and C57BL/6 mice (6–8 weeks old) were obtained from Charles River Laboratory China (Beijing, China) and maintained under specific pathogen-free conditions at a room temperature of 22 ± 2°C and air humidity of 55 ± 10% on a 12 h/12 h light/dark cycle. All protocols conformed to guidelines in Beijing University of Chinese Medicine (Beijing, China), and animal care was performed in compliance with the Principles of Laboratory Animal Care. The study was approved by the Ethical Committee on Animal Research in Beijing University of Chinese Medicine.

### Cell Isolation and Culture

The murine microglia cell line, BV-2 cells, was obtained from Beijing Union Medical University (Beijing, China) and maintained in high glucose DMEM medium supplemented with 10% heat-inactivated FBS, 100 U/ml penicillin and 100 μg/ml streptomycin at 37°C under 5% CO_2_.

Mouse marrow derived dendritic cells (BMDC) were generated as described (Varga et al., 2007). Briefly, single-cell suspensions from bone marrow of C57BL/6 mice were prepared and red blood cells were removed with lysis buffer (10 mM NH_4_Cl). Then the bone marrow cells were seeded into 6-well tissue culture plate in complete medium (RPMI1640 medium containing 10% heat-inactivated FBS, 100 U/ml penicillin and 100 μg/ml streptomycin) for 2 h at 37°C under 5% CO_2_. After the non-adherent cells were washed out, the adherent cells were cultured in the presence of 20 ng/ml GM-CSF and 10 ng/ml IL-4 for 6 days, with a medium change every second day. The yield of CD11c^+^ cells was routinely greater than 50%.

For the activation assay of neutrophils, bone marrow (BM) cells of BALB/c mice were isolated by flushing the femur BM tissues with 1% FBS-PBS, and single-cell suspension was obtained. Then the BM cells were maintained in IMDM medium supplemented with 2% heat-inactivated FBS, 100 U/ml penicillin and 100 μg/ml streptomycin at 37°C under 5% CO_2_.

Mouse spleen of BALB/c mouse was removed and single-cell suspension was generated by homogenization and passage through a 70 μm cell strainer. Red blood cells were removed with lysis buffer. The remaining splenocytes were maintained in RPMI1640 medium supplemented with 10% FBS, 100 U/ml penicillin and 100 μg/ml streptomycin at 37°C under 5% CO_2_. To purify CD4^+^ T cells, naive CD4^+^ T cells and CD8^+^ T cells, splenocytes were resuspended in 1 ml FACS buffer (PBS, 0.5% BSA, 2 mM EDTA) and isolated with negative magnetic bead separation kits of CD4^+^ T cells, naive CD4^+^ T cells, and CD8^+^ T cells according to the manufacturer’s protocols. Cell purity was analyzed using flow cytometry (>90%).

### Cytotoxicity Assay

The BV-2 cells were inoculated into 96-well plates (5 × 10^3^ cells/well). After incubated for 24 h, the cells were treated with or without GYF-21 for 24 h. Then 20 μl of reagent in CCK-8 was added into each well and incubated for 4 h according to the manufacture’s protocols. The culture plate was detected at the wavelength of 450 nm on a microplate reader. The OD values indicated the levels of cell viability.

Splenocytes were seeded in 96-well plate (1 × 10^6^ cells/well) and were treated with or without GYF-21 for 24 h. The levels of cell viability were detected as above.

### Analysis of mRNA Transcription and Secretion of Cytokines and Chemokines by Microglia

For real-time fluorescence quantitative polymerase chain reaction (PCR) assay of mRNA transcription, BV-2 cells were seeded in 6-well plate (2 × 10^5^ cells/well). After incubated for 24 h, the cells were treated with or without GYF-21 for 0.5 h and then stimulated with LPS. After stimulated 6 h by LPS, total RNA was extracted and reverse-transcribed into cDNA for 30 min at 42°C using RT kit. The cDNA was amplified by PCR Supermix kit. Sequences of the GAPDH, TNF-α,IL-6, IL-1β, MCP-1, and MIP-1α primers are shown in **Table 1**. Amplification reactions were performed for 1 cycle at 95°C for 1 min, 40 cycles at 95°C for 5 s, 60°C for 40 s, followed by 1 cycle of melting curve determination on CFX96 Touch^TM^ PCR System (BioRad, Hercules, CA, United States). The amount of mRNA in each sample was calculated and normalized to the level of GAPDH mRNA.

**Table 1:** Primer sequences used in RT-PCR.

Genes	Primer sequences
TNFα	Forward 5′-CCCCTTTATTGTCTACTCCT-3′
	Reverse 5′-AAAGCCCATTTGAGTCCTTG-3′
IL-6	Forward 5′-AAATAGTCCTTCCTACCCCAA-3′
	Reverse 5′-CCGAGTAGATCTCAAAGTGAC-3′
IL-1β	Forward 5′-GAGCCCATCCTCTGTGACTC-3′
	Reverse 5′-TCAGCTCATATGGGTCCGACA-3′
MCP-1	Forward 5′-ACCTGCTGCTACTCATTCACC-3′
	Reverse 5′-CCATTCCTTCTTGGGGTCAG-3′
MIP-1α	Forward 5′-ATTCCACGCCAATTCATCGTT-3′
	Reverse 5′-TCTGCCGGTTTCTCTTAGTCA-3′
GAPDH	Forward 5′-GCGACTTCAACAGCAACTCC-3′
	Reverse 5′-CACCCTGTTGCTGTAGCCGT-3′


For quantitative analysis of cytokine secretion, BV-2 cells were seeded in 96-well plate (2 × 10^4^ cells/well). After incubated for 24 h, the cells were treated with or without GYF-21 for 0.5 h and then stimulated with LPS. After stimulated 12 h by LPS, levels of TNF-α, IL-6, IL-1β, MCP-1, and MIP-1α in the cell medium were measured by ELISA according to the manufacture’s protocols.

### Activation Analysis of Dendritic cells

BMDC were seeded in 48-well plate (1.5 × 10^5^ cells/well). The cells were treated with or without GYF-21 for 0.5 h and then stimulated with 0.5 μg/ml LPS for 24 h. The expressions of CD80 and CD86 on dendritic cells which were gated by CD11c were determined by flow cytometry on BD FACSCanto II (BD Biosciences, San Diego, CA, United States).

### Activation Analysis of Neutrophils

Mouse BM cells were seeded in 24-well plate (1 × 10^6^ cells/well). The cells were treated with or without GYF-21 for 0.5 h and then stimulated with 20% conditioned medium (medium of BV-2 cells after stimulated with 0.5 μg/ml LPS for 24 h) for 1.5 h. The expressions of CD11b and CD62L on neutrophils which were gated by Ly-6G were determined by flow cytometry.

### Activation and Proliferation Analysis of CD4^+^ T cells

For CD4^+^ T cell activation assay, splenocytes were treated with or without GYF-21 for 0.5 h. Then the cells were seeded in 24-well plate (1 × 10^6^ cells/well) coated with anti-CD3e mAb (4 μg/ml) and stimulated with soluble anti-CD28 mAb (2 μg/ml) for 24 h. The expressions of CD69 and CD25 on the surface of CD4^+^ T cells were determined by flow cytometry.

For CD4^+^ T cell proliferation assay, purified CD4^+^ T cells were treated with or without GYF-21 for 0.5 h. Then the cells were seeded in 96-well plate (2 × 10^5^ cells/well) coated with anti-CD3e IgG (4 μg/ml) and stimulated with soluble anti-CD28 IgG (2 μg/ml) for 72 h. Then 20 μl of reagent in CCK-8 was added into each well and incubated for 4 h. The culture plate was detected at the wavelength of 450 nm on a microplate reader. The OD values were used to indicate the levels of cell proliferation.

### Differentiation Assay of CD4^+^ T Cells

Purified naive CD4^+^ T cells were treated with or without GYF-21 for 0.5 h. Then the cells were seeded in 24-well plate (5 × 10^5^ cells/well) and stimulated to differentiate into Th1 and Th17 cells. For Th1 cell differentiation under skewing conditions, naive CD4^+^ T cells were cultured with anti-CD3e mAb (4 μg/ml) plus anti-CD28 mAb (2 μg/ml) in the presence of murine IL-12 (10 ng/ml), IFN-γ (10 ng/ml), anti-mouse IL-4 mAb (10 μg/ml). To generate Th17 cells under skewing conditions, naive CD4^+^ T cells were cultured with anti-CD3e mAb (4 μg/ml) plus anti-CD28 mAb (2 μg/ml) in the presence of murine IL-6 (20 ng/ml), TGF-β (5 ng/ml), anti-IFN-γ mAb (10 μg/ml), anti-IL-4 mAb (10 μg/ml). Four days after priming, cells were washed and restimulated with PMA (50 ng/ml) and ionomycin (1 μM) for 6 h and with monensin for last 4 h, and the frequencies of Th1 and Th17 cells in live cells were determined by flow cytometry.

### Analysis of the Activation, Proliferation, and IFN-γ Secretion of CD8^+^ T Cells

For CD8^+^ T cells activation assay, splenocytes were treated with or without GYF-21 for 0.5 h. Then the cells were seeded in 24-well plate (1 × 10^6^ cells/well) coated with anti-CD3e mAb (4 μg/ml) and stimulated with soluble anti-CD28 mAb (2 μg/ml) for 24 h. The expressions of CD69 and CD25 on the surface of CD8^+^ T cells were determined by flow cytometry.

For CD8^+^ T cell proliferation and interferon-γ (IFN-γ) secretion assays, purified CD8^+^ T cells were treated with or without GYF-21 for 0.5 h. Then the cells were seeded in 96-well plate (3 × 10^5^ cells/well) coated with anti-CD3e IgG (4 μg/ml) and stimulated with soluble anti-CD28 IgG (2 μg/ml) for 72 h. Subsequently, 100 μl medium was collected from each well and the level of IFN-γ in the medium was determined by ELISA. Then 10 μl of reagent in CCK-8 was added into each well and incubated for 4 h. The culture plate was detected at the wavelength of 450 nm on a microplate reader. The OD values were used to indicate the levels of cell proliferation.

### Western Blot Analysis

BV-2 cells were seeded in 6-well plate (2 × 10^5^ cells/well). After incubated for 24 h, the cells were treated with or without GYF-21 for 0.5 h and then stimulated with LPS for 30 min or 3 h. After stimulation, the cells were collected with cell scraper and total cell proteins were extracted with RIPA lysis buffer. Then proteins were separated by 10% SDS-PAGE and transferred to polyvinylidenedifluoride membranes. The membranes were blocked in 5% skim milk for 1 h and incubated overnight at 4°C with antibodies against p65, I-κB, p38, ERK1/2, JNK, STAT1, STAT3 and their phosphorylated forms. Membranes were rinsed and incubated with horseradish peroxide-conjugated secondary antibodies for 2 h at room temperature, then rinsed again. Bands were visualized by ECL reagents on ImageQuant^TM^ LAS4000 (GE Healthcare Bio-Sciences, Pittsburgh, PA, United States).

### Statistical Analysis

All quantitative data were expressed as mean ± standard deviation (SD) of values from three independent experiments. Statistical analyses were performed with GraphPad Prism 5.0. One-way ANOVA followed by Dunnett’s test was used to determine statistical significance. A *P*-value less than 0.05 was considered to be statistically significant.

## Results

### Structural Identification and Purity Analysis of GYF-21

The structure of GYF-21 was determined to be (1a*S*, 2*S*, 3*S*, 7b*R*)-2, 3-dihydroxy-5-(4-methoxyphenethyl)-2, 3-dihydro-1a*H*-oxireno [2, 3-*f*] chromen-7 (7b*H*)-one. ^1^H NMR (CD_3_OD, 500 MHz) δ: 3.97 (1H, d, *J* = 4.0 Hz, H-5), 3.74 (1H, d, *J* = 1.0 Hz, H-6), 3.89 (1H, d, *J* = 6.5, 1.0Hz, H-7), 4.37 (1H, d, *J* = 7.0 Hz, H-8), 7.16 (2H, d, *J* = 8.5 Hz, H-2′, 6′), 6.85 (2H, d, *J* = 8.5 Hz, H-3′, 5′), 6.25 (1H, s, H-3), 3.71 (3H, s, 4′-OCH_3_), 2.89 (4H, m, H-7′, 8′). ^13^C NMR (DMSO-*d*_6_, 125 MHz) δ: 169.2 (C-2), 113.6 (C-3), 180.0 (C-4), 49.2 (C-5), 55.0 (C-6), 70.5 (C-7), 68.2 (C-8), 159.1 (C-9), 120.4 (C-10), 131.6 (C-1′), 129.2 (C-2′), 113.8 (C-3′), 157.7 (C-4′), 113.8 (C-5′), 129.2 (C-6′), 30.9 (C-7′), 34.1 (C-8′), 55.5 (4′-OCH_3_). ESI-MS: *m/z*: 331 [M + H]^+^, molecular formula: C_18_H_18_O_6_. All spectral data were in agreement with literature data (Wu et al., 2012). The purity of GYF-21 was greater than 94% according to HPLC analysis on LCMS-IT-TOF platform (**Figure 1B**).

### Cytotoxicity of GYF-21 on Microglia and Splenocytes

**FIGURE 2:**
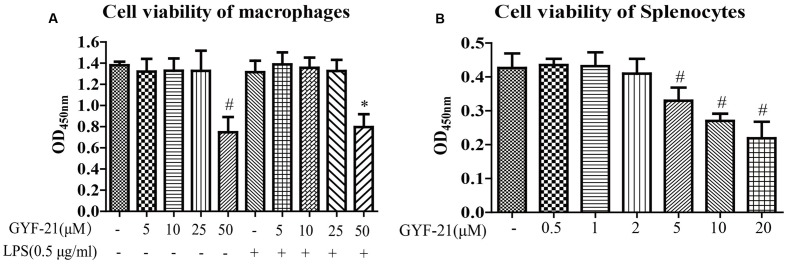
**Cytotoxicity of GYF-21 on microglia and splenocytes.** BV-2 cells **(A)** and splenocytes **(B)** were treated with various concentrations of GYF-21 for 24 h and cell viability was determined with CCK-8. Data are representative of three independent experiments. ^#^*P* < 0.05 vs. Vehicle; ^∗^*P* < 0.05 vs. LPS.

In order to investigate the immunosuppressive effects of GYF-21 under doses without cytotoxicity, the cytotoxicity of GYF-21 on microglia and splenocytes was evaluated with CCK-8. The results (**Figure 2**) showed that GYF-21 had no cellular toxicity on BV-2 cells at the concentration up to 25 μM and on splenocytes at the concentration up to 2 μM.

### Inhibitory Effects of GYF-21 on the Expression of Cytokines and Chemokines by Microglia

**FIGURE 3:**
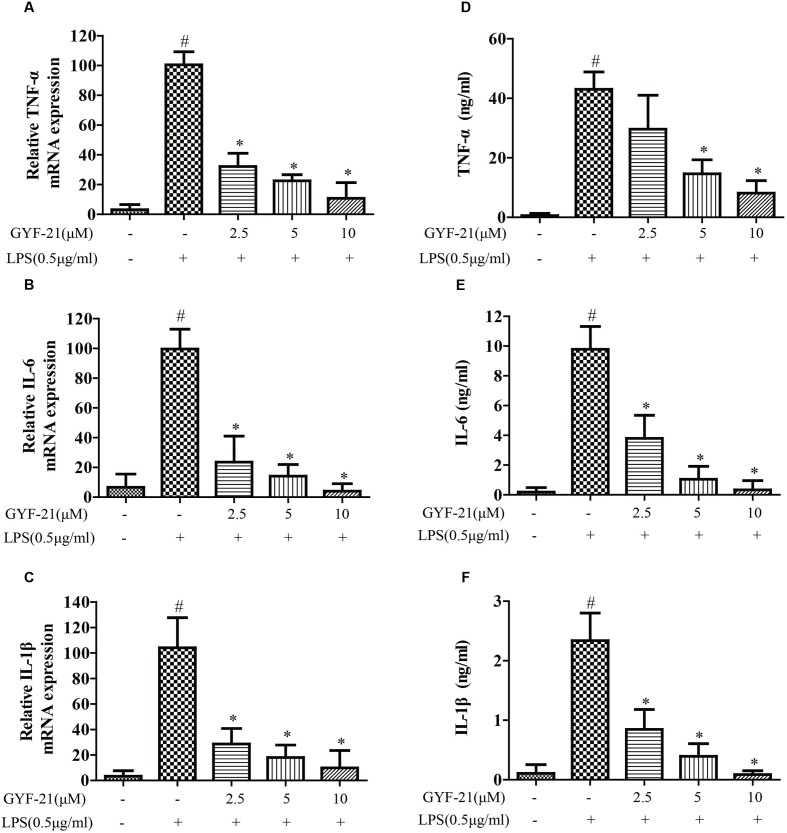
**Effects of GYF-21 on the expression of pro-inflammatory cytokines by microglia.** BV-2 cells were pretreated with various concentrations of GYF-21 for 0.5 h and then stimulated with LPS (0.5 μg/ml) for 6 h (RT-PCR) or 12 h (ELISA). The mRNA transcription of TNF-α, IL-6, and IL-1β was determined by RT-PCR **(A–C)**. The levels of TNF-α, IL-6, and IL-1β in the supernatants were determined by ELISA **(D–F)**. Data are representative of three independent experiments. ^#^*P* < 0.05 vs. Vehicle, ^∗^*P* < 0.05 vs. LPS.

**FIGURE 4:**
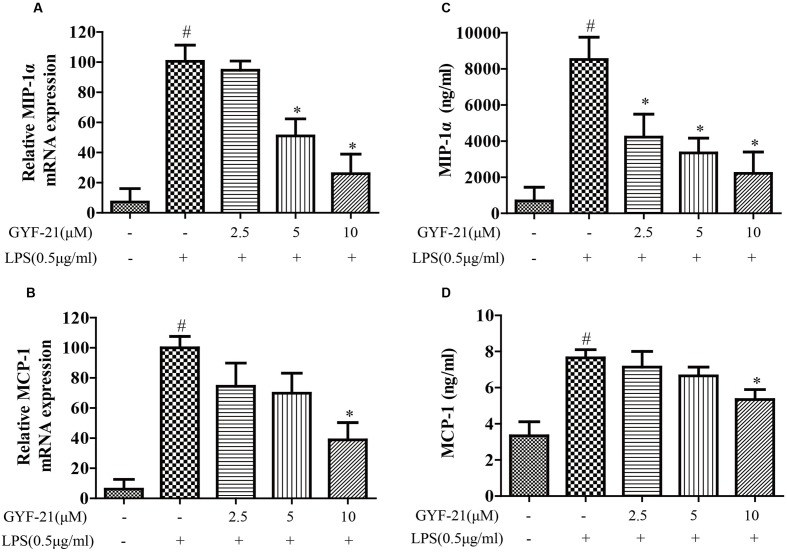
**Effects of GYF-21 on the expression of chemokines by microglia.** BV-2 cells were pretreated with various concentrations of GYF-21 for 0.5 h and then stimulated with LPS (0.5 μg/ml) for 6 h (RT-PCR) or 12 h (ELISA). The mRNA transcription of MIP-1α and MCP-1 was determined by RT-PCR **(A,B)**. The levels of MIP-1α and MCP-1 in the supernatants were determined by ELISA **(C,D)**. Data are representative of three independent experiments. ^#^*P* < 0.05 vs. Vehicle, ^∗^*P* < 0.05 vs. LPS.

The overproductions of pro-inflammatory cytokines and chemokines by microglia, such as TNF-α, IL-6, IL-1β, MCP-1, and MIP-1α, play important roles in the pathogenesis of MS (Ogura et al., 2008; Starossom et al., 2012). To investigate the effects of GYF-21 on innate immune cells, the effects of GYF-21 on the expression of cytokines and chemokines by LPS induced microglia were detected firstly. As shown in **Figures 3**, **4**, compared to the control group, the mRNA transcription and protein secretion of pro-inflammatory cytokines and chemokines, TNF-α, IL-6, IL-1β, MCP-1, and MIP-1α, were both dramatically elevated in LPS-treated group. However, GYF-21 intensively inhibited the expressions of TNF-α, IL-6, and IL-1β in a dose-dependent manner (inhibition rates of 60–99%) and moderately suppressed the expressions of MCP-1and MIP-1α (inhibition rate of 40 or 80% for high dose treatment).

### Effects of GYF-21 on the Activation of Dendritic Cells

**FIGURE 5:**
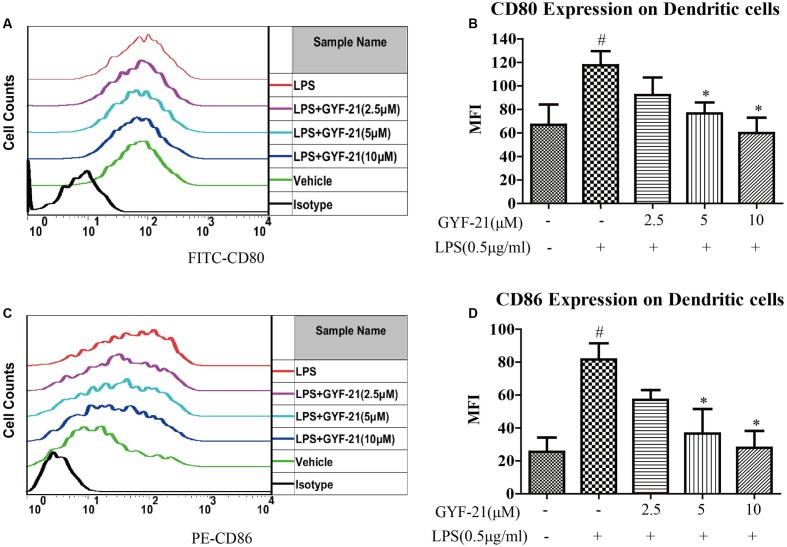
**Effects of GYF-21 on the activation of dendritic cells.** BMDC were pretreated with various concentrations of GYF-21 for 0.5 h and then stimulated with LPS for 24 h. The expression of CD80 **(A,B)** and CD86 **(C,D)** on BMDC (CD11c^+^ cells) were determined by flow cytometry. Data are representative of three independent experiments. ^#^*P* < 0.05 vs. Vehicle, ^∗^*P* < 0.05 vs. LPS.

Dendritic cells are professional antigen presenting cells and link the innate and adaptive immune system. When dendritic cells are activated, CD80 and CD86 on dendritic cells upregulated rapidly and provide costimulatory signal necessary for T cell activation and survival (Wu and Laufer, 2007; Comabella et al., 2010; Nuyts et al., 2013). After demonstrated the inhibitory effects of GYF-21 on the activation of microglia, we further investigated the effects of GYF-21 on the activation of dendritic cells. The results (**Figure 5**) showed that GYF-21 significantly suppressed upregulation of CD80 and CD86 on CD11c^+^ dendritic cells in a dose-dependent manner (inhibition rates of 40–80%).

### Effects of GYF-21 on the Activation of Neutrophils

**FIGURE 6:**
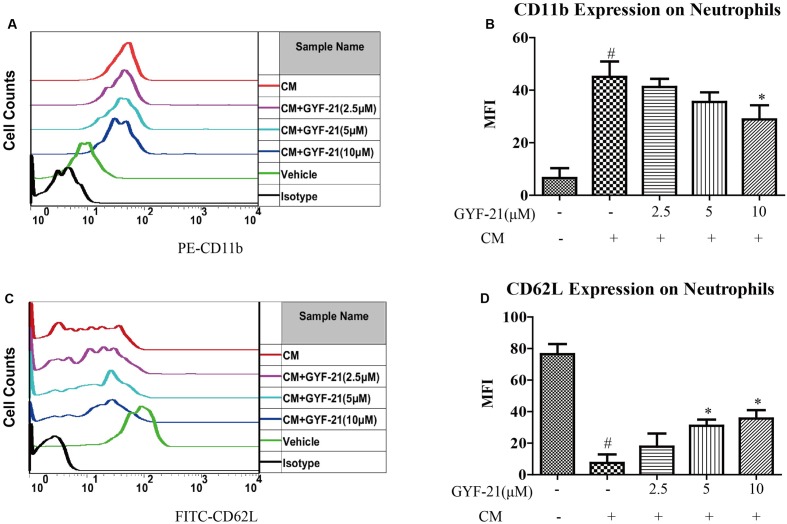
**Effects of GYF-21 on the activation of neutrophils.** BM cells were pretreated with various concentrations of GYF-21 for 0.5 h and then stimulated with conditioned medium for 1.5 h. The levels of CD11b **(A,B)** and CD62L **(C,D)** on neutrophils (Ly-6G^+^ cells) were determined by flow cytometry. Data are representative of three independent experiments. ^#^*P* < 0.05 vs. Vehicle, ^∗^*P* < 0.05 vs. CM.

Neutrophils are the most abundant type of granulocytes and the most abundant type of white blood cells in most mammals. They also form an essential part in the pathogenesis of MS (Steinbach et al., 2013). After discovered the inhibitory effects of GYF-21 on the activation of microglia and dendritic cells, we also investigated the effects of GYF-21 on the activation of neutrophils. The results (**Figure 6**) showed that GYF-21 slightly suppressed conditioned medium-induced CD11b upregulation and CD62L shedding on the Ly-6G^+^ neutrophils in a dose-dependent manner (inhibition rates of 15–30%).

### Effects of GYF-21 on the Activation of NF-κB, MAPK, and STAT1/3 Signaling Pathways in Microglia

**FIGURE 7:**
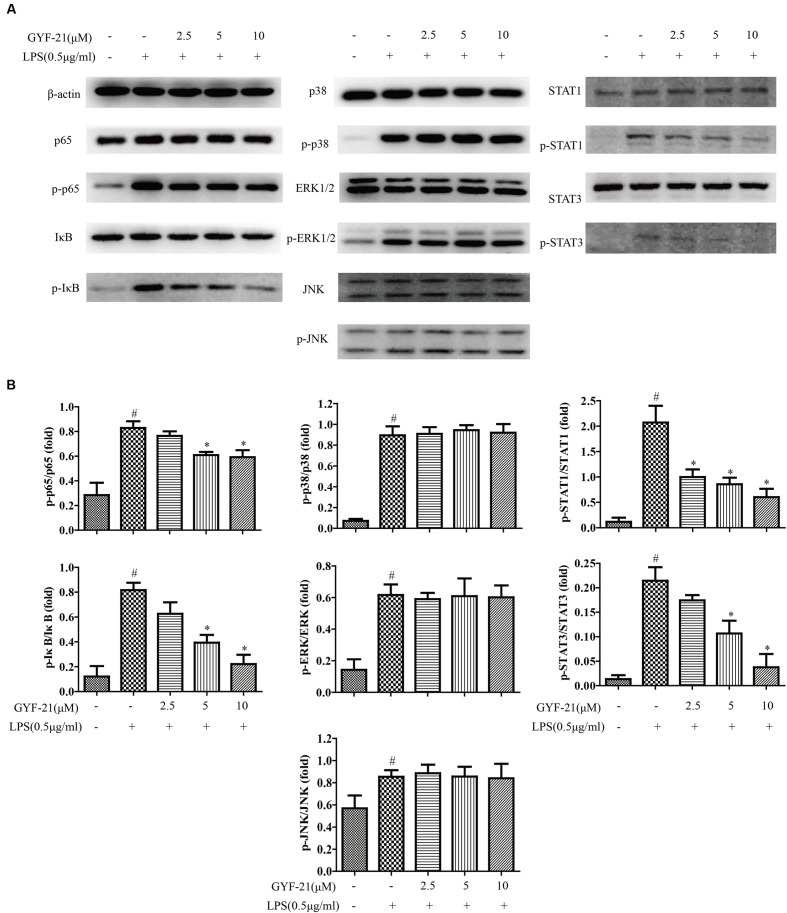
**Effects of GYF-21 on the activation of NF-κB, MAPK, and STAT1/3 signaling pathways.** BV-2 cells were pretreated with various concentrations of GYF-21 for 0.5 h and then stimulated with LPS (0.5 μg/ml) for 0.5 or 3 h. The expressions of p65, I-κB, p38, ERK1/2, JNK, STAT1, STAT3 and their phosphorylated forms were determined by western blot **(A,B)**. A typical result in three independent experiments is shown. ^#^*P* < 0.05 vs. Vehicle, ^∗^*P* < 0.05 vs. LPS alone.

It is well-known that nuclear factor kappa B (NF-κB), mitogen-activated protein kinase (MAPK), and signal transducer and activator of transcription 1/3 (STAT1/3) signaling pathways play critical roles in the function regulation of innate immune cells (Tak and Firestein, 2001; Li and Verma, 2002; Shuai and Liu, 2003; Zhang and Dong, 2005; Miklossy et al., 2013; Schwartz et al., 2015). In order to reveal the mechanism of GYF-21 inhibiting the activation of innate immune cells, we investigated the effects of GYF-21 on the activation of NF-κB, MAPK and STAT1/3 signaling pathways in microglia induced by LPS. The results (**Figure 7**) showed that highly phosphorylation of p65, I-κB, p38, ERK1/2, JNK, STAT1, and STAT3 was observed in LPS-induced BV-2 cells and GYF-21 significantly inhibited the phosphorylation of STAT1/3, p65 and I-κB in a dose-dependent manner rather than the phosphorylation of p38, ERK1/2, and JNK.

### Effects of GYF-21 on the Activation and Proliferation of CD4^+^ T Cells

**FIGURE 8:**
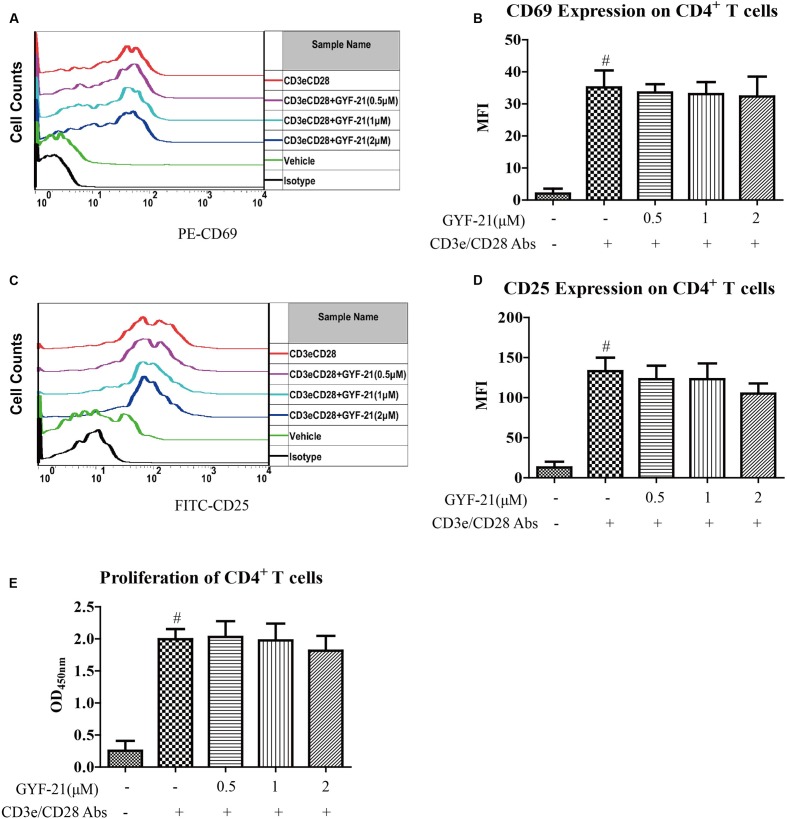
**Effects of GYF-21 on the activation and proliferation of CD4^+^ T cells.** Splenocytes were pretreated with or without GYF-21 for 0.5 h. Then the cells were seeded in 24-well plate (1 × 10^6^ cells/well) coated with anti-mouse CD3e IgG (4 μg/ml), and stimulated with anti-mouse CD28 IgG (2 μg/ml) for 24 h. The levels of CD69 and CD25 on CD 4^+^ T cells were determined by flow cytometry **(A–D)**. CD4^+^ T cells were purified by negative magnetic bead isolation kit and pretreated with or without GYF-21 for 0.5 h. Then the cells were seeded in 96-well plate (2 × 10^5^ cells/well) coated with anti-mouse CD3e IgG (4 μg/ml) and stimulated with anti-mouse CD28 IgG (2 μg/ml) for 72 h. CD4^+^ T cell proliferation was determined with CCK-8 **(E)**. Data are representative of three independent experiments. ^#^*P* < 0.05 vs. Vehicle, ^∗^*P* < 0.05 vs. CD3e/CD28 Abs.

After discovered the inhibitory effects of GYF-21 on the activation of multiple innate immune cells via suppressing STAT1/3 and NF-κB signaling pathways, we furtherly investigated the effects of GYF-21 on adaptive immunity in virtue of important roles that STAT1/3 and NF-κB signaling pathways play in adaptive immunity. Firstly, we investigated the effects of GYF-21 on the activation and proliferation of CD4^+^ T cells. The results (**Figure 8**) showed that GYF-21 didn’t suppress upregulation of CD69 and CD25 on CD4^+^ T cells and proliferation of CD4^+^ T cells stimulated by anti-mouse CD3e and CD28 IgG.

### Effects of GYF-21 on the Differentiation of CD4^+^ T Cells

**FIGURE 9:**
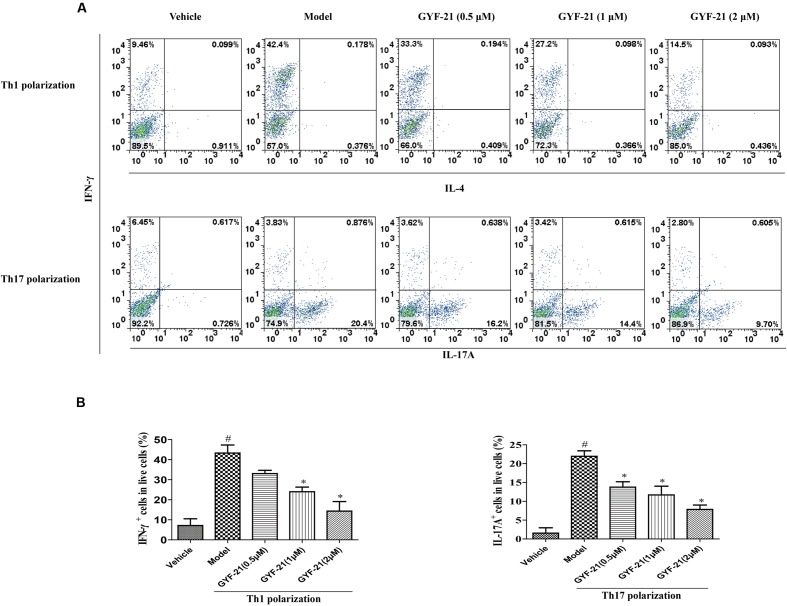
**Effects of GYF-21 on the differentiation of CD4^+^ T cells.** Purified naive CD4^+^ T cells were treated with or without GYF-21 for 0.5 h. Then the cells were seeded in 24-well plate (5 × 10^5^ cells/well) and stimulated to differentiate into Th1 and Th17 cells. On day 4 after priming, cells were washed and stimulated again with PMA (50 ng/ml) and ionomycin (1 μM) for 6 h and with monensin (2 μM) for last 4 h. The frequencies of Th1 and Th17 cells in live cells were determined by flow cytometry **(A,B)**. Data are representative of three independent experiments. ^#^*P* < 0.05 vs. Vehicle, ^∗^*P* < 0.05 vs. Model.

CD4^+^ T cells are a type of T cell that plays central role in the pathogenesis of MS. In the presence of cytokines produced by cells of innate immunity, naive CD4^+^ T cells differentiate into helper T cells subset with different functions and cytokine profiles. Three main helper T cell subsets, Th1, Th2, and Th17, were described. Previous studies demonstrated that both Th1 cells and Th17 cells were able to independently induce MS. After determined the inhibitory effects of GYF-21 on the activation of STAT1/3 signaling pathways which play major roles in promoting CD4^+^ T cells to differentiate into Th1 and Th17 cells respectively, we further investigated the effects of GYF-21 on the differentiation of naive CD4^+^ T cells. The results (**Figure 9**) showed that GYF-21 significantly inhibited the differentiation of naive CD4^+^ T cells into Th1 and Th17 cells in a dose-dependent manner (inhibition rates of 30–75%).

### Effects of GYF-21 on the Activation, Proliferation, and IFN-γ Secretion of CD8^+^ T Cells

**FIGURE 10:**
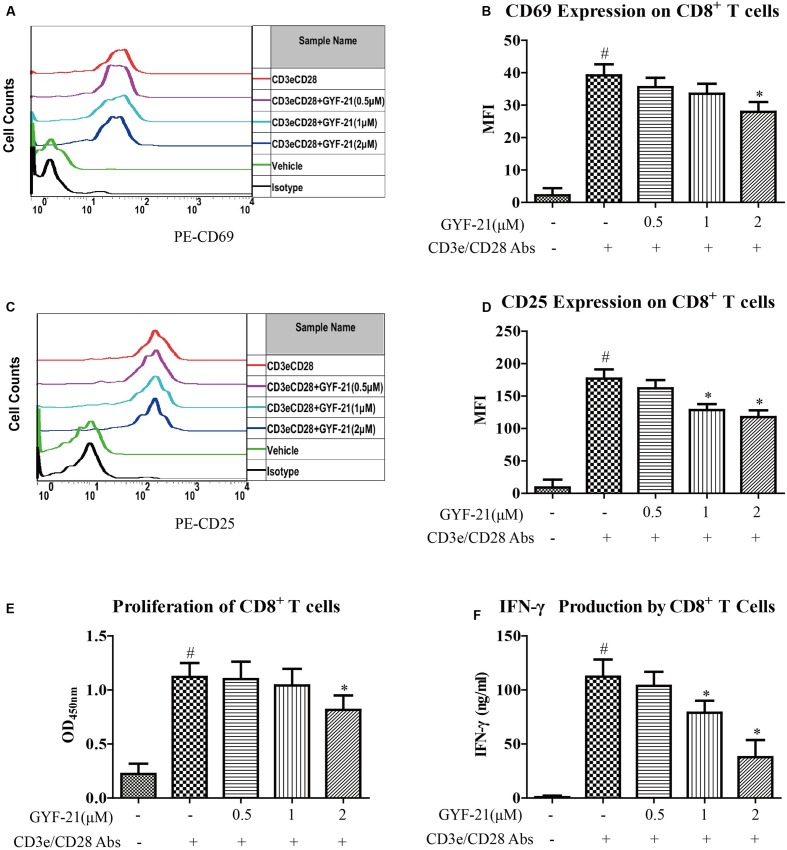
**Effects of GYF-21 on the activation, proliferation, and IFN-γ secretion of CD8^+^ T cells.** Splenocytes were treated with or without GYF-21 for 0.5 h. Then the cells were seeded in 24-well plate (1 × 10^6^ cells/well) coated with anti-CD3e mAb (4 μg/ml) and stimulated with soluble anti-CD28 mAb (2 μg/ml) for 24 h. The expressions of CD69 and CD25 on the surface of CD8^+^ T cells were determined by flow cytometry **(A–D)**. Purified CD8^+^ T cells were treated with or without GYF-21 for 0.5 h. Then the cells were seeded in 96-well plate (3 × 10^5^ cells/well) coated with anti-CD3e IgG (4 μg/ml) and stimulated with soluble anti-CD28 IgG (2 μg/ml) for 72 h. Subsequently, the proliferation of CD8^+^ T cells was determined with CCK-8 **(E)**. The level of IFN-γ in the medium was determined by ELISA **(F)**. Data are representative of three independent experiments. ^#^*P* < 0.05 vs. Vehicle, ^∗^*P* < 0.05 vs. CD3e/CD28 Abs.

The mechanisms studied in MS focus on the activity of myelin-specific CD4^+^ T cells. However, CD8^+^ T cells are also strongly implicated in the pathogenesis of MS (Ji et al., 2013). After determined the inhibitory effects of GYF-21 on the differentiation of CD4^+^ T cells into Th1 cells, it’s meaningful to further investigate the effects of GYF-21 on CD8^+^ T cells. Then we determined the effects of GYF-21 on the activation, proliferation, and IFN-γ secretion of CD8^+^ T cells. The results (**Figure 10**) showed that GYF-21 slightly suppressed upregulation of CD69 and CD25 on CD8^+^ T cells and proliferation of CD8^+^ T cells stimulated by anti-mouse CD3e and CD28 IgG (inhibition rates of 15–30% for high dose treatment). In addition, GYF-21 moderately inhibited IFN-γ secretion of CD8^+^ T cells in a dose-dependent manner (inhibition rates of 0–60%).

## Discussion

Microglia, dendritic cells, and neutrophils are major innate immune cells that play important roles in pathogenesis of MS. Microglia are resident tissue macrophages located in the CNS. The interactions between microglia and T cells are crucial in MS pathobiology (Starossom et al., 2012; Goldmann and Prinz, 2013; Strachan-Whaley et al., 2014). Dendritic cells are the primary antigen presenting cells directing T cell function and are extremely important in directing the immune pathology characteristic of MS (Wu and Laufer, 2007; Comabella et al., 2010; Nuyts et al., 2013). Neutrophils are required for the maturation of myeloid cells into professional APCs and local restimulation of myelin-specific T cells (Jaillon et al., 2013; Steinbach et al., 2013).

In this study, we firstly determined the inhibitory effects of GYF-21 on the activation of microglia cell line BV-2 induced by LPS. The results showed that GYF-21 intensively inhibited the expression of pro-inflammatory cytokines, TNF-α, IL-6 and IL-1β, and moderately suppressed the expression of chemokines, MCP-1 and MIP-1α. After demonstrated the inhibitory activity of GYF-21 on the activation of microglia, we investigated the effects of GYF-21 on the activation of dendritic cells and neutrophils. The results showed that GYF-21 significantly inhibited upregulation of costimulatory molecules, CD80 and CD86, on dendritic cells. In addition, GYF-21 also slightly inhibited upregulation of CD11b and shedding of CD62L on neutrophils. These data revealed the inhibitory effects of GYF-21 on the activation of microglia, dendritic cells and neutrophils with different strengths, and suggested that GYF-21 could significantly inhibit innate immunity with combined inhibitory effects on above innate immune cells.

NF-κB, MAPK, and STAT1/3 signaling pathways play key roles in signal transduction during innate immune responses (Tak and Firestein, 2001; Li and Verma, 2002; Shuai and Liu, 2003; O’Shea et al., 2004; Zhang and Dong, 2005; Loo et al., 2006; Schulz et al., 2011; Miklossy et al., 2013; Schwartz et al., 2015). After revealed the inhibitory effects of GYF-21 on the activation of innate immunity, we investigated its mechanism by monitoring the activation of NF-κB, MAPK, and STAT1/3 signaling pathways in BV-2 cells induced by LPS. The results showed that GYF-21 significantly suppressed the activation of STAT1/3 and NF-κB signaling pathways but not MAPK signaling pathways. These data indicated that GYF-21 inhibited innate immunity through blocking STAT1/3 and NF-κB signaling pathways. In view of important roles that STAT1 and STAT3 signaling pathways play in the production of Th1 and Th17 cells, GYF-21 may be able to inhibit the differentiation of CD4^+^ T cells into Th1 and Th17 cells.

CD4^+^ T cells are central roles in regulating adaptive immunity responses. Once activated, these cells expanded and differentiated into different Th subsets with distinct cytokine profiles and effector functions. The main Th subsets are Th1, Th2, and Th17 cells. Previous studies have revealed that Th1 and Th17 cells are involved and play key roles in pathogenesis of MS (Ogura et al., 2008; McGeachy et al., 2009; Lazarevic et al., 2011; Goldmann and Prinz, 2013; Martin et al., 2016). Recent years, some studies have also reported that CD8^+^ T cells are strongly implicated in the pathogenesis of MS (Ji et al., 2013).

After we revealed the inhibitory effects of GYF-21 on STAT1/3 signaling pathways, we firstly investigated the effects of GYF-21 on the activation and proliferation of CD4^+^ T cells. The results showed that GYF-21 didn’t suppress upregulation of CD69 and CD25 on CD4^+^ T cells and following proliferation of CD4^+^ T cells. Then we further investigated the effects of GYF-21 on the differentiation of naive CD4^+^ T cells. The results showed that GYF-21 intensively inhibited the differentiation of naive CD4^+^ T cells into Th1 and Th17 cells. In addition, further study revealed that GYF-21 could also slightly suppress activation and proliferation of CD8^+^ T cells, and moderately inhibited IFN-γ secretion of CD8^+^ T cells. These data suggest that GYF-21 may be potential to treat MS by inhibiting adaptive immune function.

In particular, many studies have demonstrated that Th17 cells play most important roles in pathogenesis of MS. The initial development of Th17 cells from naive CD4^+^ T cells is directed by TGF-β in combination with IL-6, and this process is enhanced by IL-1β and TNF-α, whereas IL-23 is required for the terminal differentiation of Th17 cells into mature effector cells (Ogura et al., 2008; McGeachy et al., 2009; El-Behi et al., 2011; Martin et al., 2016). The present study demonstrated that GYF-21 not only directly inhibited differentiation of naive CD4^+^ T cells into Th17 cells by blocking IL-6-STAT3 and IL-23-STAT3 signaling pathways but also intensively reduced production of TNF-α, IL-6, and IL-1β by microglia. These data suggests that GYF-21 is highly valuable to be used for treating MS through significantly inhibiting production of Th17 cells. In our previous study, we had reported a chloride substituted 2-(2-phenethyl)-chromone (GYF-17) with immunosuppressive activity which had similar structure with GYF-21 and marked inhibitory activity on STAT1/3 (main mechanism) and ERK1/2 signaling pathways (Zhu et al., 2016). So we propose that GYF-21 exerts inhibitory effects on STAT1/3 signaling pathways directly but not resulted from downstream response of inhibitory effects on NF-κB signaling pathway. Furthermore, it’s well-known that activation of NF-κB signaling pathway induces stronger immunological response than ERK1/2 signaling pathway. As well, NF-κB signaling pathway exerts apoptosis promoting effects, and ERK1/2 signaling pathway play key role in promoting cell proliferation. So double inhibitory effects of GYF-21 on STAT1/3 and NF-κB signaling pathways make it to exhibit stronger immunosuppressive effects and lower cytotoxicity than GYF-17.

## Conclusion

In summary, we have discovered that GYF-21, an epoxide 2-(2-phenethyl)- chromone derivative isolated from Chinese agarwood, can significantly suppress innate and adaptive immunity via inhibiting STAT1/3 and NF-κB signaling pathways and exhibits great potential to be developed into therapeutic agent for MS. Meanwhile, the therapeutic applications of GYF-21 *in vivo* and the internal association of inhibitory effects of GYF-21 on STAT1/3 and that on NF-κB signaling pathways need further study.

## Author Contributions

Z-XZ initiated the project and wrote the paper. Z-XZ, RG, Y-FZ, Y-FG, H-XH, and S-SL performed the experiments and analyzed the data. P-FT and Y-LS participated in study design and coordination as well as drafted and revised the manuscript. All authors read and approved the final manuscript.

## Conflict of Interest Statement

The authors declare that the research was conducted in the absence of any commercial or financial relationships that could be construed as a potential conflict of interest.
